# The EU Health Union in Search of a Definition and an Open Discussion

**DOI:** 10.1007/s10272-021-0971-z

**Published:** 2021-06-04

**Authors:** Agnes Sipiczki, Karel Lannoo

**Affiliations:** Centre for European Policy Studies (CEPS), 1 Place du Congrès, 1000 Brussels, Belgium

## Abstract

More than other EU ‘Unions’, the proposed Health Union requires proper definition because the EU’s competences are limited in this domain.

More than other EU 'Unions', the proposed Health Union requires proper definition because the EU's competences are limited in this domain. The COVID-19 crisis has highlighted the hazardous inefficiency of piecemeal national responses. It has also shown that individual member states cannot tackle the challenges of cross-border health threats alone.

Creating more unity in public health policy will be an uphill battle, however. Sensitivities about privacy and the General Data Protection Regulation (GDPR) are a major barrier, even at the national level. A 'taxonomy' is therefore needed to gather comparable EU-wide data held in national systems.

For the pharmaceutical sector, the vaccine debate has shown that the lack of a single capital market and delays to a truly single patent law or a single EU legal framework are major stumbling blocks, even if the industry is essential to Europe's economy.

## Upgrading existing agencies while creating a new one

The European Commission is rapidly advancing in the public health space, but a proper analysis of the room for manoeuvre and parameters, for example within the treaties, has not yet been carried out. The Commission has proposed an upgrade of its core agencies, the European Medicines Agency (EMA) and the European Centre for Disease Prevention and Control (ECDC), and the creation of a new one: the European Health Emergency Preparedness and Response Authority (HERA). It has increased the amount of funds available for health research in the Multiannual Financial Framework (2021-27) under different budget lines and is starting to adopt a harder stance towards the pharma industry in meeting public demands. But the policy expertise comes from the member state or local level, and the data required for good policymaking is missing at all levels. The GDPR hampers the collection and sharing of European citizens' health data; healthcare systems' data are not standardised, and aggregate data on R&D at the public and private levels are not available for national or for EU use.

Strictly speaking, the EU only has a supplementary and coordinating competence in public health policy because it is a core member state or regional responsibility. With the EMA, the European Commission has shaped the Single Market with a common commercial framework for healthcare products, covering intellectual property, R&D support and open markets. The EU can intervene in emergency situations to combat major cross-border health threats (on the basis of Article 168(5) of the Treaty on the Functioning of the European Union, TFEU) and stimulate cross-border cooperation. Yet the Commission did not make joint advance purchases of COVID-19 vaccines on this basis; rather, it relied on an emergency support mechanism designed for humanitarian assistance in the event of natural disasters (Article 122 TFEU).

Advancing further without treaty amendments or an open debate about competences, as the Commission is doing implicitly, has raised a number of problems that demonstrate the importance of a more carefully planned expansion in the area of public health. The Commission is creating expectations that are difficult to fulfil given the limited expertise, data, means and capacity in this domain. It cannot realise a Health Union without a proper democratic process and the express political support of member states. A more open debate is therefore required, without hasty moves that may backfire or bring the Union's legal or structural limitations to the fore.

The Commission is using the urgency of the health crisis as an argument to advance amendments to the EMA and ECDC regulations (as stated, without carrying out an in-depth impact assessment) and to create HERA. Both the

* This contribution draws on a report for a Healthcare and Pharmaceuticals Working Group of the CEPS Industrial Policy Task Force.

**Agnes Sipiczki**, Centre for European Policy Studies, Brussels, Belgium; and European University Institute, Florence, Italy.

**Karel Lannoo**, Centre for European Policy Studies, Brussels, Belgium; and European University Institute, Florence, Italy.

EMA and ECDC will receive additional tasks and responsibilities under the draft regulations, which, in the case of the ECDC, should have started with a proper fact-finding inquiry into why the agency was entirely behind the curve of the pandemic in early 2020. This could reveal problems of competence and related issues of inadequate data. This same question could be raised for HERA: due to the fact that it is very difficult to gather data and member states are unwilling to share competences in healthcare, should a thorough assessment be required before creating a new agency? An in-depth debate on these matters can avoid future problems (and one that goes beyond the online consultation that ran until 12 May).

## The vaccine debate

The joint procurement by the EU of COVID-19 vaccines through Advance Purchase Agreements (APAs) was announced already in August 2020. The procurement process is run by the Commission on behalf of all participating member states, following a 2013 EU decision, with a budget of €2.7 billion (Decision No 1082/2013/EU, 2013).[1] The liability for the deployment and use of the vaccine remains with the purchasing EU member states. The ensuing discussion, above all in early 2021, however, raised many questions about the negotiation tactics used by the EU Commission, the price setting and the lack of transparency. The impression emerged that the Commission had limited expertise in dealing with large pharma groups, and in the understanding of the business models, as evidenced by the way the UK, even though it is dependent on imports, managed to get better deals and a quicker delivery. This led to the adoption of an export authorisation scheme (Commission Implementing Regulation (EU) 2021/111, 2021) by the EU in January 2021, which has caused a great deal of controversy. The EU's intention is to ensure that European citizens get access to vaccines that have been funded by EU money, and that pharmaceutical companies respect their contractual commitments. But instead, the situation raised the spectre of industry re-localisations out of the EU for an industry that is the main EU exporter.

The EU agreed upon prices with manufacturers, which vary with a factor of eight. The Belgian state secretary for the budget mistakenly released the prices agreed with manufacturers (see Table 1), which make one wonder why the EU paid so much to certain manufacturers in the first place. One would expect much smaller price differences in a competitive market, or more flexible prices. Price elasticity could be expected to be high, certainly in the early days of the vaccination, and would be a good incentive for producers. This pricing agreement, however, excluded it. Additionally, leaving the liability with the member states is an unclear and unworkable structure.

Table 1
**Vaccines prices and quantity for Belgium**

| Company | Price in € | Number (in mn) |
|---|---|---|
| AstraZeneca | 1.78 | 7.70 |
| Johnson & Johnson | 7 | 5.17 |
| Sanofi-GSK | 7.56 | 7.74 |
| Pfizer-BioNTech | 12 | 5.10 |
| Curevac | 10 | 5.80 |
| Moderna | 15 | 2.10 |

Source: Belgian state secretary for the budget, December 2020.

The recent European Commission (2021) proposal for Digital Green Certificates, which would facilitate the free movement of vaccinated or otherwise protected (i.e. recovered) persons by creating an interoperable framework for vaccine certificates, also raises a number of sensitive questions about the collection, processing and retention of digital health data. It is essential to ensure the sufficient protection of citizens' personal data on their vaccination status or previous COVID-19 infections, as the Secretary General of the Council of Europe, Marija Pejčinović Burić, reminded the 47 member states in a recent statement (Council of Europe, 2021). According to the proposal, this data would be stored on the certificates and would be accessible to national authorities across the Union. During the negotiations, the Council proposed including further details related to the collection of personal data, such as the possibility of it being processed for purposes other than exercising free movement rights.

## A pharma sector policy

Similar concerns arise around the EU's pharma sector strategy, for which much more data of a macro and micro nature is needed. The European Commission wants to have more say over big pharma but lacks information and understanding of the industry. Given the critical importance of pharma and its participation in publicly funded research, consolidated information on the strengths and weaknesses of the industry, and its structure and orientation should be publicly available. The EU should have an aggregate view of all the forms of R&D support for the sector at the national, European and international levels. This would allow for better targeted public spending in

1 A proposal to update the 2013 decision was presented by the European Commission (2020) on 11 November 2020.

this domain. Today, it is unevenly spread over many different programmes or is uncoordinated. To our knowledge, nobody has the aggregate picture.

The competitiveness of the pharma sector in Europe is conditioned by other factors that have languished on the EU's agenda. Indeed, the COVID-19 crisis has highlighted the urgent need to increase the resilience of pharmaceutical and healthcare supply chains and to achieve some strategic autonomy in the sector. Europe still has no single patent law or single capital market – two factors crucially important for pharma and biotech companies – and lags far behind the US in its number of patents, while China is catching up rapidly. The problem is the lack of a unitary patent, or the presence of a hybrid model in which national and Union law coexist. A Unified Patent Court for the EU is only starting to function. This increases the costs for biotech firms and reduces the solidity of research frameworks.

On the capital markets side, the lack of a single market sends all successful biotech companies to the US, to the extent that 98% of follow-up offerings by European biotech firms have been on US rather than European exchanges (Le Deu and Santos da Silva, 2019). The broader problem is again the lack of a truly single legal framework with strong enforcement. The lack of a unitary patent system also prevents a quick decision over an EU-wide waiver of intellectual property rights for COVID-19 vaccines, as is being discussed now.

Europe has been successful in its public-private partnerships to stimulate R&D in pharma, but the amounts involved are one-tenth of what is spent by the US Biomedical Advanced Research and Development Authority, and there is insufficient follow-up and coordination between the European and national R&D programmes. Increasing research and innovation capacity in Europe's pharmaceutical sector, combined with a more robust production capacity, would not only make the industry more competitive globally, it would also strengthen the EU's capacity to manage future pandemics and health threats.

The next seven year EU budget (2021-27) increased core health R&D funding from €7.4 billion to €8 billion, but more is available through other budget lines. The last Horizon programme (2014-20) had assigned €2.1 billion for the funding programme in the domain of vaccines, through vaccination research and through support for the Innovative Medicines Initiative (IMI), which is co-funded 50%-50% with the private sector. The new MFF has a budget of €3.6 billion for IMI and its successor, the Innovative Health Initiative, in order to create an EU-wide health research and innovation ecosystem that facilitates the translation of scientific knowledge into tangible innovations.

The new EU budget has enhanced facilities for supporting equity financing and risk capital of high growth enterprises. This will help prevent strong European biotech firms from moving to the US for the next stage of funding. The EU has addressed this problem in the InvestEU programme (the Juncker plan), which also includes budget lines for public-private partnerships in biotech, and has been amplified by the von der Leyen Commission as well as under the new EU budget. The new budget also has a strong increase for the EU4Health programme, with a budget of €1.8 billion to strengthen health systems and the healthcare workforce, to support integrated and coordinated work between the EU member states, to offer a sustained promotion of the implementation of best practices in (global) data sharing, to reinforce the healthcare workforce and to tackle the implications of demographic challenges.

## The road ahead

For a Health Union to work, a candid and ambitious debate is needed on what the EU wants, how the competences will be shared with member states, and how it will improve on all the deficiencies revealed during this crisis. More data is needed to formulate a better view on where the EU stands in public health policy and in health research. The sensitive issue of personal health data must also be addressed. More unity is needed in intellectual property regulation and in capital markets. A long-term agenda needs to be set without hasty decisions and with a proper debate, which is an issue that can be raised at the Future of Europe conference.
